# A novel selection strategy for antibody producing hybridoma cells based on a new transgenic fusion cell line

**DOI:** 10.1038/s41598-020-58571-w

**Published:** 2020-02-03

**Authors:** Martin Listek, Anja Hönow, Manfred Gossen, Katja Hanack

**Affiliations:** 1 Immunotechnology Group, Institute of Biochemistry and Biology, University of Potsdam, Potsdam, Germany; 2 Berlin-Brandenburg Center for Regenerative Therapies (BCRT), Berlin, Germany; 3 Helmholtz-Zentrum Geesthacht, Institute of Biomaterial Science, Teltow, Germany; 4 new/era/mabs GmbH, August-Bebel-Str. 89, Potsdam, Germany

**Keywords:** Antibody generation, Assay systems

## Abstract

The use of monoclonal antibodies is ubiquitous in science and biomedicine but the generation and validation process of antibodies is nevertheless complicated and time-consuming. To address these issues we developed a novel selective technology based on an artificial cell surface construct by which secreted antibodies were connected to the corresponding hybridoma cell when they possess the desired antigen-specificity. Further the system enables the selection of desired isotypes and the screening for potential cross-reactivities in the same context. For the design of the construct we combined the transmembrane domain of the EGF-receptor with a hemagglutinin epitope and a biotin acceptor peptide and performed a transposon-mediated transfection of myeloma cell lines. The stably transfected myeloma cell line was used for the generation of hybridoma cells and an antigen- and isotype-specific screening method was established. The system has been validated for globular protein antigens as well as for haptens and enables a fast and early stage selection and validation of monoclonal antibodies in one step.

## Introduction

**Figure 1:**
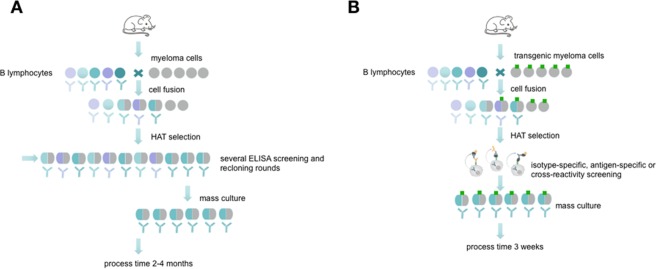
Schematic overview about conventional hybridoma technology compared side by side to the new selection approach. The picture (reprinted by permission from Springer Nature^10^) shows the process of monoclonal antibody generation via conventional hybridoma technology (**A**) and via the new selection approach using transgenic fusion cell lines (**B**). The fusion with transgenic myeloma cells allows a fast and efficient hybridoma screening in an isotype- or antigen-specific manner and allows an early screening for possible cross-reactivities.

Antibodies are well known as universal binding molecules with a high specificity for their corresponding antigens and have found, therefore, widespread use in very many different areas of biology and medicine^1^. Most murine antibodies are produced today by means of the hybridoma technique as monoclonal antibodies^2^ or with the help of antibody gene libraries and display techniques as recombinant antibody fragments^3^. Both methods have intrinsic advantages but also difficulties such that they are restricted to specialized laboratories and companies. Currently, the reliability of monoclonal antibodies was critically discussed in several publications^4,5^ which is related to a growing demand of better validation and characterization of these molecules^6–8^. Especially the hybridoma technique which results in full-length monoclonal antibodies can be cumbersome, labour-intensive and time-consuming (Fig. 1A). Although several improvements have been tried in the course of the past years, the basic method is still very similar to the original method published by Köhler and Milstein^9–11^. The critical issue in the development of antigen-specific hybridomas is the lack of any direct connection between the hybridoma cell and the released antibody. Therefore, it is necessary to perform limited dilution techniques in order to separate single cells to ensure monoclonality. Unfortunately, this process could not be combined with a simultaneous, proper validation of the desired antibodies because the concentration in the supernatants are often very low at the early beginning of culture.

To facilitate the isolation of specific antibody-producing hybridomas, a method has to be established which temporarily restricts the cells from releasing the antibody into the culture medium and thus retaining the genotype (the antibody-coding genes) and the phenotype (the produced antibodies) in one entity. Such precondition can easily be fulfilled when recombinant antibody fragments are isolated, e.g. by phage display techniques. To confer this basic principle to the hybridoma technique would require to capture the synthesized antibody on the surface of the synthesizing hybridoma cell (Fig. 1B). To realize this, a covalent surface labeling of antibody-producing cells with biotin was accomplished in the past, which allowed the isolation of specific cells by means of avidin- or streptavidin-conjugated ligands binding the released antibodies^12^. However, chemical surface labeling is very often unpredictable and may disturb normal functions and the vitality of the cells.

**Figure 2:**
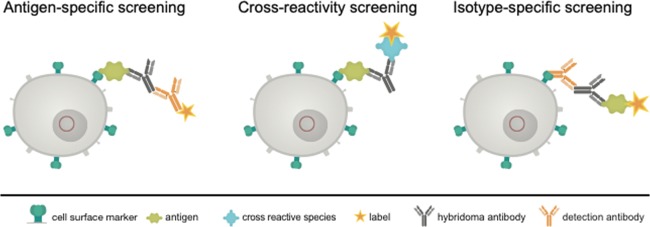
Schematic view of the proposed selection principle. Shown is a transgenic hybridoma cell line (in grey) with an artificial marker construct (HA-AP-EGF-R, in dark green) present on the cell surface. The genetic construct (red circle) contains a truncated variant of the human immature EGF-receptor (EGF-R), a hemagglutinin epitope (HA) and a biotin acceptor peptide (AP). The secreted hybridoma antibody (black) can be linked to the corresponding cell by binding to the antigen (light green) or to an isotype-specific detection antibody either (orange). Sorting of specific hybridomas is performed by using appropriate labels conjugated to a secondary antibody or to the antigen of interest.

We, therefore, tried to replace this principle by a more gentle method. We transfected the myeloma cells to be used for hybridoma fusion with a construct enabling the expression of a surface marker containing the acceptor peptide (AP) sequence for site-specific biotinylation by biotin ligase (BirA)^13^. Such a surface marker, after *in vitro* biotinylation, should be applicable for the isolation of antigen-specific antibody-producing hybridoma, allowing for a built-up of a bridge e.g. with the streptavidin-conjugated antigen or isotype-specific antibody, which in turn catches the produced antibody, and a labeled indicator anti-immunoglobulin or antigen (Fig. 2). The system allows a combination of three possible sorting options. The antigen-specific approach (Fig. 2, left) is performed by an antigen-avidin complex bound to the biotinylated cell. The antigen is specifically recognized by the secreted antibody and the detection takes place via a secondary antibody labelled to a fluorescent dye. This approach can be extended to a cross-reactivity screening (Fig. 2, middle panel), where different antigen-avidin complexes can be linked to the cell surface and the secreted antibodies can be tested for a specific binding. This approach is also transferable to the isotype-specific approach shown in Fig. 2 on the right panel. Here, an isotype-specific antibody, such as an anti-IgG antibody, coupled to avidin, is linked to the cell surface. The secreted antibody, in case it is an IgG, is caught and the dye-coupled antigen is used for fluorescence detection. In dependance of the antigen and the selection principle all three options can be combined or performed consecutively. This principle allows a fast and specific sorting of antigen-specific hybridoma cells 10 days after HAT selection and avoids laborious limited dilution techniques and ELISA screenings.

In order to realize this principle a suitable gene construct was designed and transfected into myeloma cells to establish a cell line stably expressing the construct on the cell surface. The next steps were to prove that the expression pattern did not change significantly after fusion of the transfected myelomas with B lymphocytes and that the system can indeed be used to isolate specific antibody-producing hybridomas. The results shown here prove that an easy and efficient selection of specific antibody-producing cells is possible with this novel method.

## Results

### HA-AP-EGF-R expression on transfected myeloma cells

**Figure 3:**
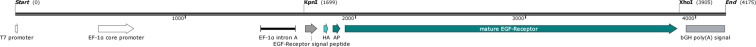
Vector design of the artificial cell surface receptor. To express the HA-AP-EGF-receptor fusion protein on the surface of myeloma cells, the signal peptide of the immature human EGF-receptor was inserted at the N-terminus of the cloned hemagglutinin epitope (HA) containing a biotin acceptor peptide (AP) sequence, and a truncated variant of the mature humane EGF-receptor (aa 1-651) at its C-terminus. The construct is controlled by the EF1α-promoter.

**Figure 4:**
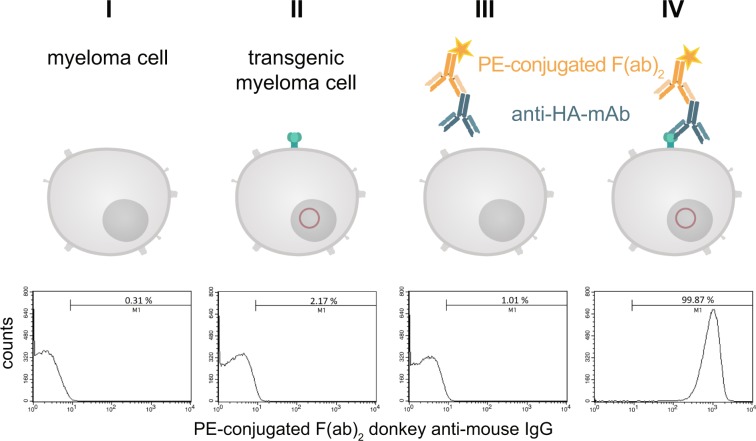
Detection of the HA-tag on the cell surface of transfected myeloma cells. Stable transfected and non-transfected myeloma cells (1 × 10^6^) were stained with a monoclonal anti-HA.11 antibody and a PE-labeled F(ab)_2_ fragment of a donkey anti-mouse IgG. After staining and washing the cells were analyzed by flow cytometry. Non-stained cells served as negative control. To exclude non-living cells a staining with 7-AAD was performed. The histograms represent all living cells from this approach with 1 × 10^5^ recorded events. Analysis was performed by using BD CellQuest Pro software version 6.0.

The construct to be used for transfection (Fig. 3) contained the signal peptide of the immature human EGF-R followed by the hemagglutinin epitope (HA) containing the biotin acceptor peptide (AP) and the extracellular domain and transmembrane domain of the mature human EGF-R (aa 1-651). The elements were chosen because the EGF-R is one of the best characterized receptors in literature and it is known which truncated versions still provide a faithful transmembrane localisation, while being devoid of signalling activity. The latter is important to prevent unwanted interference with intracellular signalling upon ectopic transgene expression^14–16^. The HA epitope was used as detection element to visualize the marker on the surface of the cells and the AP sequence is necessary for the biotinylation. The transfection of myeloma cells performed by transposase-mediated gene transfer resulted in stable expression of HA-AP-EGF-R on the cell surface. This could be shown by a monoclonal anti-HA.11 antibody and a phycoerythrin (PE)-labeled F(ab)_2_ fragment of a donkey anti-mouse IgG in flow cytometry experiments. Over 99% of the transfected cells could be positively stained for the artificial cell surface construct (Fig. 4IV).

**Figure 5:**
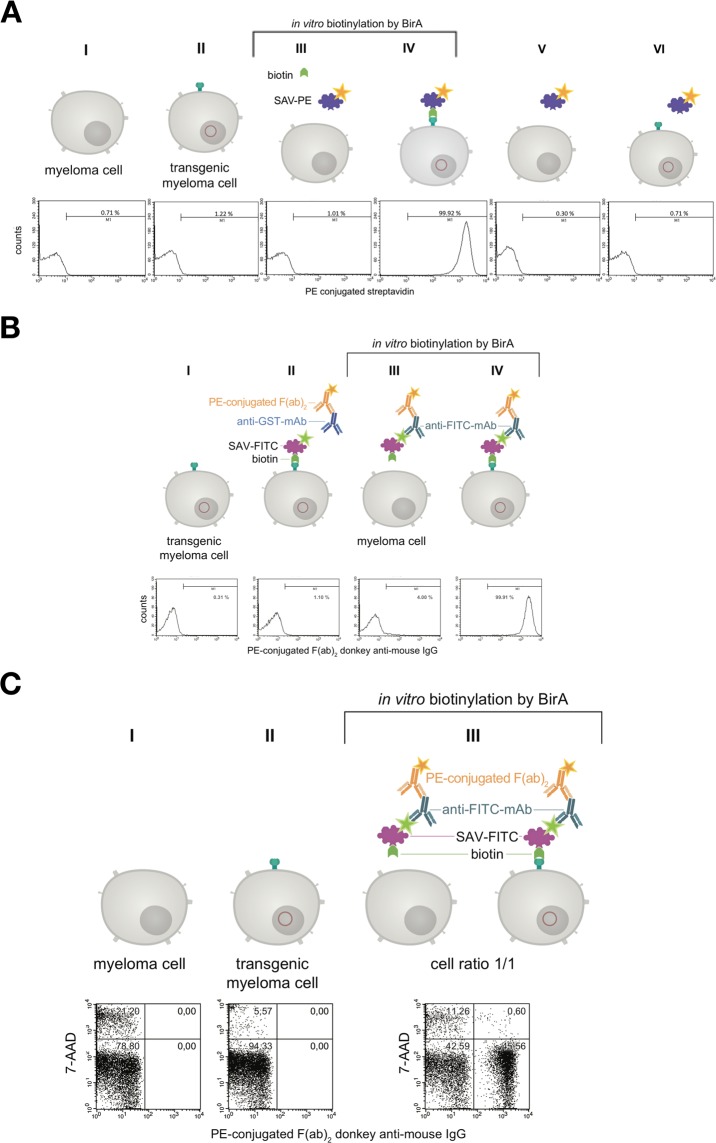
Characterization of the artificial receptor functions on myeloma cells. (**A**) The biotin acceptor peptide (AP) is accessible on the surface of stably transfected myeloma cells. Transfected and non-transfected myeloma cells (1 × 10^6^) were used for BirA mediated biotinylation. The cells in approaches III & IV were incubated with a mixture consisting of 5 mM MgCl_2_, 1 mM ATP, 10 µM Biotin and 0.789 µg BirA. The cells in approaches I, II, V, VI were incubated without BirA. To investigate whether BirA mediated biotinylation reaction was successful and specific, the cells (III, IV, V, VI) were treated with PE-conjugated streptavidin (2 µg/mL). After staining and washing cells were analyzed and 2.5 × 10^4^ events were recorded by flow cytometry. Non-stained cell served as negative control. Dead cells were excluded by 7-AAD-staining. Analysis was performed by using BD CellQuest Pro software version 6.0. (**B**) The artificial receptor as bridge between phenotype and genotype. To investigate whether the HA-AP-EGF-R is a suitable tool to retain a soluble antibody at the surface of biotinylated transfected cells, a stable bridge was constructed between the receptor, an antigen and the antibody. Fluorescein was used as antigen which was bound to the surface as FITC-labeled SAV (2 µg/mL). The monoclonal mouse anti-fluorescein antibody B13-DE1 was used as antibody to be bound. As an isotype control a mouse anti-GST antibody (1 µg/1 × 10^6^ cells) was used. A PE-conjugated F(ab)_2_ fragment of a donkey anti-mouse IgG diluted in PBS containing 0.5% BSA and 2 mM EDTA was used as indicator antibody. Dead cells were excluded by staining with 7-AAD and 1 × 10^4^ events were recorded. Analysis was performed by using BD CellQuest Pro software version 6.0. (**C**) Antigen loaded HA-AP-EGF-R+ myeloma cells can be identified within a heterogeneous cell population. Transfected and non-transfected myeloma cells (1 × 10^6^) were mixed together in a cell ratio of 1/1 followed by *in vitro* biotinylation reaction. The cells from approach III were incubated with a mixture of 5 mM MgCl_2_, 1 mM ATP, 10 µM Biotin and 0.789 µg BirA. The cells from approaches I and II were incubated without BirA. Following that the heterogeneous cell pool was incubated with a FITC-labeled SAV-conjugate (2 µg/mL). After this, the cells were labeled with B13-DE1 (1 µg/1 × 10^6^ cells) and a PE-conjugated F(ab)_2_ fragment of a donkey anti-mouse IgG. Non-stained cells served as negative control and dead cells were excluded by staining with 7-AAD. 1 × 10^4^ events were recorded and analysed by using BD CellQuest Pro software version 6.0.

In the next step the binding of PE-conjugated streptavidin (SAV) to the myeloma cell surface after biotinylating the artificial surface marker was verified by flow cytometry as shown in Fig. 5A. Over 99% of cell population IV showed a positive staining compared to non-transfected control cells. When using fluorescein isothiocyanate (FITC)-conjugated SAV, the mouse monoclonal anti-FITC antibody B13-DE1 and a PE-labeled F(ab)_2_ fragment of a donkey anti-mouse IgG, over 99% of the cells could be stained (Fig. 5B). After receptor-expressing and non-expressing myeloma cells were mixed in equal numbers and the same treatment was performed, i.e. FITC-conjugated SAV, mouse monoclonal anti-FITC antibody B13-DE1 and PE-labeled F(ab)_2_ fragment (donkey anti-mouse IgG), about 45% of the cells were stained, which was the expected ratio used in the initial experiment (Fig. 5C). These experiments showed clearly the specific labeling of the cells. The same results were obtained when using ovalbumin-conjugated SAV, external monoclonal anti-ovalbumin antibodies and PE-labeled F(ab)_2_ fragment of a donkey anti-mouse IgG (data not shown) showing that it is feasible to apply the system both for haptens as well as globular proteins.

**Figure 6:**
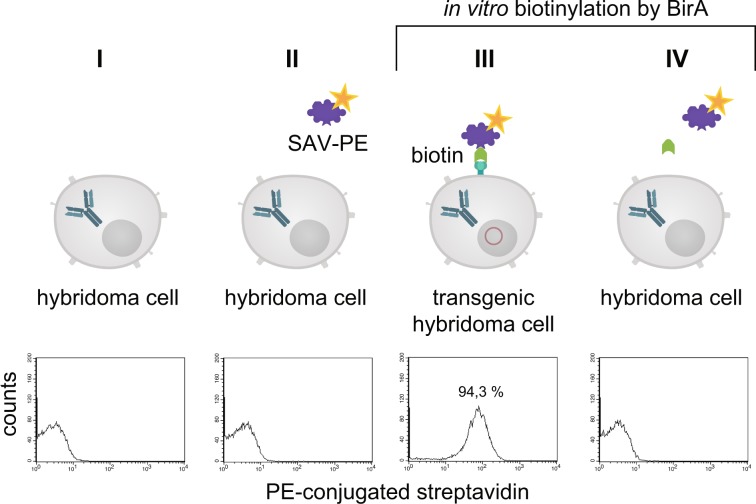
The artificial cell surface receptor is present after cell fusion and HAT selection on the surface of hybridoma cells. Transgenic and non-transgenic hybridoma cells (1 × 10^6^) were used for *in vitro* biotinylation. The cells from approach III and IV were incubated with a mixture of 5 mM MgCl_2_, 1 mM ATP, 10 µM Biotin and 0.789 µg BirA. The cells from approach I and II were incubated without BirA. BirA mediated biotinylation was screened by treating the cells (III, IV) with PE-conjugated streptavidin (2 µg/mL). After staining and washing the cells were analyzed by flow cytometry. Non-stained cell served as negative control. Dead cells were excluded by staining with 7-AAD. 2 × 10^4^ events were recorded and analysed by using BD CellQuest Pro software version 6.0.

**Figure 7:**
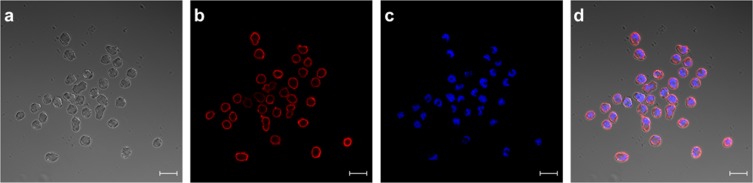
Staining of HA-AP-EGF-R bearing hybridoma cells. HA-AP-EGF-R bearing hybridoma cells were stained for receptor expression using PE-conjugated streptavidin. DAPI-staining was performed in panel c. Both stainings were merged in panel d together with panel a which represents the differential interference contrast picture. Immunofluorescence pictures were taken with an LSM 880 (Carl Zeiss, Germany) with a 20 µm size bar.

In the next experiments we showed, that the receptor expression did not cease after fusing the transfected myeloma cells with lymphocytes from mice (Fig. 6, Panel III). In order to visualize receptor expression on biotinylated hybridoma cells fluorescence was performed with PE-conjugated SAV (Fig. 7). Panel 7d showed a merge of differential interference contrast, DAPI- and PE-conjugated SAV staining which presents the expected stained surface corona on all cells imaged.

### Selection of ovalbumin-specific hybridomas

**Figure 8:**
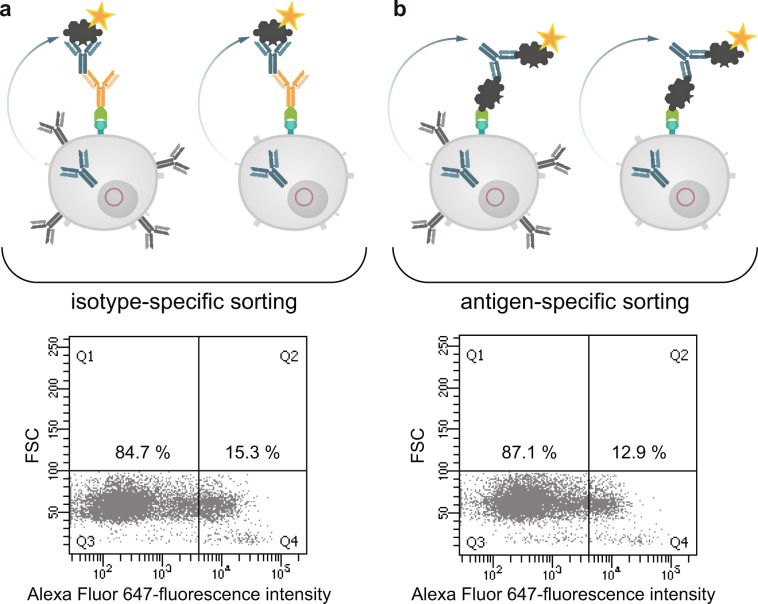
Isotype-specific as well as antigen-specific labeling of antibody producing hybridomas. Antibody producing HA-AP-EGF-R+ hybridoma cells (1 × 10^6^) were biotinylated *in vitro* by BirA and incubated with an avidin-conjugated goat anti-mouse IgG specific antibody (panel a) or with ovalbumin conjugated to avidin (panel b) for 20 min at 4 °C. For antibody production and secretion the hybridoma cells were washed (PBS, pH 7.4, 0.5% BSA and 2 mM EDTA at 300 × g for 10 min) and incubated in full growth media for 3 h at 37 °C and 6% CO_2_. After incubation the cells were washed by using PBS, pH 7.4, 0.5% BSA and 2 mM EDTA at 300 × g for 10 min and incubated with an Alexa Fluor 647 conjugated ovalbumin (10 µg/1 × 10^6^ cells) for 20 min at 4 °C as indicator of antigen-specific captured antibodies. Dead cells were excluded by 7-AAD staining. 1 × 10^4^ events were recorded and analysed by using BD FACSDiva version 8.0.

The results revealed that the new fusion cell line should be suitable to identify antigen-specific hybridomas by cell sorting. In order to prove this directly, we performed an ovalbumin-specific fusion and combined an isotype- and antigen-specific sorting two weeks after fusion. Figure 8a shows the corresponding dot plot of hybridoma cells sorted with the isotype-specific approach. Out of 1 × 10^6^ cells from the fusion pool and after gating it was possible to stain 15.3% of the cells specifically with the isotype-specific approach which corresponds to a cell number of 49,000 cells. With the antigen-specific approach 12.9% (41,500 cells) of the cells could be stained specifically (Fig. 8b). Positive sorted cells from both sortings were exemplarily single-cell plated in one 96-well plate and tested by ELISA. We could detect an ovalbumin-specific signal in each cavity showing outgrowth of sorted hybridoma cells (data not shown).

**Figure 9:**
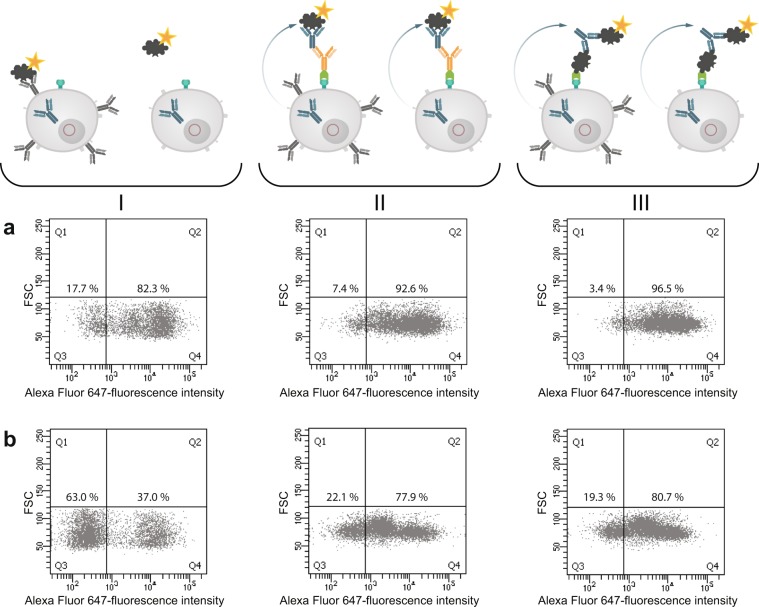
Comparison of three different sorting options for selecting specific hybridoma cells. Hybridoma cells sorted isotype-specific (panel a) and antigen-specific (panel b) were analyzed again with (I) fluorescent labeled ovalbumin, (II) isotype-specific and (III) antigen-specific via HA-AP-EGF-R. Panel I shows the results when the cells were incubated with Alexa Fluor 647 conjugated ovalbumin (10 µg/1 × 10^6^ cells) alone without using the HA-AP-EGF-R construct. In Panel (II,III) the isotype- and antigen-specific sorting was performed as described. Positive cells were displayed in Q4. Dead cells were excluded by 7-AAD staining. 1 × 10^4^ events were recorded and analysed by using BD FACSDiva version 8.0.

**Figure 10:**
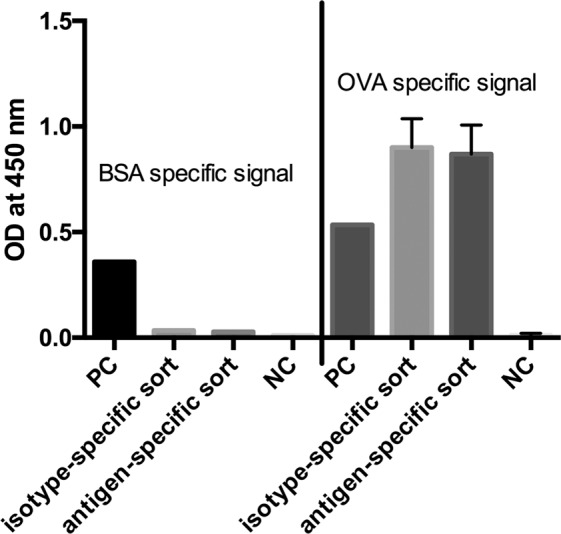
Indirect ELISA for ovalbumin and BSA specificity. Culture supernatants of the isotype- and antigen-specific approach were tested for specific antibody production by immobilizing ovalbumin and BSA to a microtiter plate (10 µg/mL). The detection of specific antibody responses was performed by an HRP-conjugated goat anti-mouse IgG antibody and TMB as substrate. Absorbance was measured at 450 nm with a reference at 630 nm. Values are given as single measurement for positive controls (PC), BSA-specific signals and negative control (NC) for BSA. Ovalbumin specific signals and NC for ovalbumin were performed twofold. Error bars represent standard deviation.

In the next step we analyzed both cell pools again with different sorting options after one week of culture (Fig. 9). In order to see wether the B cell receptor (BCR) itself is influencing the sorting procedure we labelled the cells with antigen only. In panel I fluorescently labeled ovalbumin was used to identify antigen-specific BCR + hybridomas. In the isotype-specific approach (Fig. 9a) 82.3% of the cells could be stained specifically whereas 17.7% were negative in binding the antigen (panel Ia). For cells sorted with the antigen-specific approach before we obtained a different result (Fig. 9b). Here, 63% of the population were negative in binding fluorescently labeled ovalbumin whereas only 37% were positive (panel Ib). This indicates, that nearly two thirds of the population do not carry an antigen-specific BCR on the cell surface and these could not be detected with just labelled antigen. In comparison to panel IIb and IIIb where 78% and 80% of the cells could be selected positive, the outcome of specific hybridomas could be doubled by using our new approach. Also for the isotype-specific staining it was obvious that the outcome of positive hybridomas could be further increased from 82.3% to 96.5%. These results showed that a specific labelling process independent from the BCR on the cell surface is more reliable and allows a subsequent selection of antigen-specific hybridomas already two weeks after fusion. It is well known, that the BCR itself is not presented stably on the cell surface and that internalization after polyvalent antigen binding is a common event^17^. In a further experiment, the culture supernatants of both sortings were tested in an indirect ELISA for ovalbumin specificity and cross-reactivity to bovine serum albumine (BSA) (Fig. 10). In contrast to the specific signal for ovalbumin no cross-reactive binding could be detected for BSA.

## Conclusions

In this article we describe a novel and highly effective method for the selection of antigen-specific hybridoma cells based on a new transgenic myeloma cell line. The cell line carries an artificial cell surface marker for isotype- and antigen-specific binding of produced antibodies and enables therefore a smart and fast selection and validation procedure for antigen-specific hybridomas. The field of monoclonal antibody generation via hybridoma technology is still plagued by long screening processes and suboptimal selection methods^18^ and it is rarely possible to validate selected antibodies at an early stage. A further issue is that the outcome of antigen-specific B lymphocytes depends on the individual host immune response and the composition of the immunizing antigen. This can dramatically influence the process afterwards. However, the third aspect in this field is the complex process of cell fusion and stable growth of hybridomas as such. With an input of approximately 8 × 10^7^ cells prior fusion and an outcome of 10–20 hybridomas producing a suitable monoclonal antibody this process seems to be one of the most ineffective processes known in biotechnology (efficiency of 0.000025%). In order to address this unsatisfactory situation and to find a method which can provide an efficient screening and an early validation of antibodies we developed a system for linking the genotype and the phenotype of the cell which leads to an efficient linking of the produced antibody to the corresponding hybridoma cell. With this system it is possible to simultaneously handle all positive hybridomas after fusion and to screen for antigen- and isotype-specificity in parallel. Further, the system can be flexibly used to screen e.g. for cross-reactivities when the antigen is replaced by other targets of interest.

This antigen-specific hybridoma selection reminds of the method proposed by Manz *et al*.^12^ but avoids the chemical cell surface labeling, so that the method described here will be much more reliable and easier to be performed. There are alternative selection methods available as described earlier by Browne *et al*. such as LEAP (laser-enabled analysis and processing technology)^19,20^, gel microdrop technology^21^, semisolid media^22^ or droplet based microfluidic assays^23,24^ but all these methods require an encapsulation of cells which leads to physical stress and low efficiencies with regard to high throughput production of monoclonal antibodies. Selection methods for antibody-producing hybridoma cells based on flow cytometry are described since 1979^25^ and are used today effectively with some improvements as reviewed in^26^. Nevertheless, with the exception of the droplet based assays these methods often require hybridoma cells which express the antigen-specific BCR on the cell surface in order to label the corresponding cell. Depending on the B cell differentiation at the time point of spleen cell isolation for fusion it is often the case that the generated hybridoma cells do not express BCRs anymore and so these selection methods are not suitable for those hybridomas even when they secrete highly specific antibody molecules. Further, there are several studies published where it could be shown that BCR expression depends on culture medium conditions and cell numbers^27^, cell cycle^28,29^ or secretion intensity^30^. When staining with just labelled antigen alone we could select cells but these underwent apoptosis after sorting (data not shown). Therefore, sorting via the BCR itself seems not a feasible method to identify antigen-specific hybridomas. Another method published by Pierce *et al*.^31^ described a transgenic myeloma cell line expressing Igα and Igß on the cell surface, so that the membrane-bound form of the secreted antibody could be caught and used for a flow cytometry based screening. This system is also dependent on the B cell status at the time of fusion and could lead to BCR-clustering and internalization and therefore could induce cell death as described by Li *et al*.^32^. Our developed approach is independent on BCR expression and is therefore suitable for all hybridomas generated by the fusion process. We could show that the method is applicable for low- and high-molecular weight substances such as FITC and ovalbumin, so that the basis for anti-hapten antibody and anti-protein antibody selections can be provided. Of course, the question raised, if negative cells were positively stained when secreted antigen-specific antibodies bound to the surface receptor of a neighboring cell. We have investigated different secretion temperatures and time periods and found an optimum at 37 °C for 3 h. We could not detect any significant labeling of non-producers. Further, we could transfer this selection principle to HEK293 and CHO cells in order to allow an efficient screening of cells expressing recombinant proteins and introduced BirA into the vector construct, so that the cells are able to biotinylate themselves (data not shown). The described technology in this study allows a fast and efficient identification of desired antibody-producing cells and a flexible validation with regard to isotype-specificity and cross-reactivities. In ongoing studies the focus is shifted to different classes of antigens such as transfected cells, nucleic acids or whole microorganisms to show the general feasibility of the system.

## Methods

### Cell line and cell culture

Murine non-secreting myeloma Sp2/0 cells (ATCC CRL-1581) were cultured under 6% CO_2_, at 37 °C and 95% humidity by using DMEM full growth media supplemented with 10% fetal calf serum (FCS), 2 mM glutamine, 50 µM ß-mercaptoethanol and 0.1 mg/mL gentamycine.

### Design of the surface marker gene construct HA-AP-EGF-R

To express the HA-AP-EGF-receptor fusion protein on the surface of the Sp2/0 mammalian cells, we inserted the signal peptide of the immature human EGF-receptor at the N-terminus of the cloned hemagglutinin epitope (HA) containing a biotin acceptor peptide (AP) sequence, and a truncated variant of the mature human EGF-receptor (aa 1-651) at its C-terminus (Fig. 3). The EGF receptor signal peptide (forward primer 5′ATATAGGTACCGCCACCATGCGACCCTCCG 3′ KpnI, reverse primer 5′ATTATAAGCTTAGACGAGCCTTCCTCCAGAGCC 3′ HindIII), the HA-AP sequence (forward primer 5′CATGAAAGCTTGGCTCGTCTGGGTATCCATATGATG 3′ Hind III, reverse primer 5′CTAATGGTAGCCGGCGCGCCCTCG 3′ NheI) as well as the whole extracellular domain and transmembrane domain of the mature human EGF-receptor (forward primer 5′ATAGAGCTACCGGAAGCAGCGGGCTGGAGGAAAAGA 3′ NheI, reverse primer 5′CATAAGCGGCCGCTTACCGAACGATGTGG 3′ NotI) were amplified by standard PCR techniques from the pcDNA3.1 EGF-R-YFP plasmid (this was a kind gift of Stefan Kubick, Fraunhofer Institute for Biomedical Engineering, Potsdam-Golm) and the pDisplay AP-CFP-TM plasmid (this was a kind gift of A. Ting, Department of Chemistry Massachusetts Institute of Technology, Massachusetts). Following that, the DNA fragments were cloned into the mammalian expression vector pCEP4 (Invitrogen, Paisley). Finally, the whole gene construct (HA-AP-EGF-R) was subcloned into a pPB EF1α vector^33^, such that expression of the artificial receptor from the piggyBac transposon is under control of the human elongation factor 1 alpha promoter. The integration of the HA-AP-EGF-receptor fusion gene into the vector was confirmed by sequencing (GATC Biotech AG, Konstanz). The signal peptide cleavage efficiency was determined by using the SignalP 4.0 model^34^ and the membrane topology of the fusion protein was analyzed and predicted by using HMMTOP^35,36^.

### Generation of stably HA-AP-EGF-R-transfected Sp2/0 cells

Two days prior transfection, 1.5 × 10^4^ living Sp2/0 cells were plated into each well of a 6-well plate and cultured as described above. At the day of transfection the culture supernatant was discarded and replaced by 2 mL of freshly prepared culture medium and the culture dish was further incubated at 37 °C under 6% CO_2_. The transfection with the surface marker-expressing gene construct HA-AP-EGF-R was conducted according to Kuroda *et al*.^37^ and Cardinos and Bradley^38^. Briefly, to obtain a special ratio between donor and helper plasmid, 2 µg of the plasmid pPB EF1a HA-AP-EGF-R (donor plasmid) were supplemented with 0.2 µg of the transposase plasmid (mPB, helper plasmid) and filled up to 100 µL with 150 mM NaCl (plasmid DNA mixture). Moreover 8 µL of PEI (polyethylenimin) stock solution (7.5 mM in water, pH 7.4) were diluted in 92 µL 150 mM NaCl (PEI mixture). After 10 min incubation at room temperature (RT) the PEI mixture was added to the plasmid DNA mixture and further incubated at RT for 10 min. The solution was added dropwise to the cells while gently shaking the plate. The cells were then incubated for 24 h at 37 °C under 6% CO_2_. After this treatment, the culture supernatant was replaced by fresh growth medium and the cells were cultured for another 24 h at 37 °C under 6% CO_2_. The growth medium was then exchanged by selection medium (DMEM with 10% (v/v) FCS, 2 mM glutamine, 50 µM ß-mercaptoethanol, 0.1 mg/mL gentamycine and 25 µg/mL puromycin). After 7 days of puromycin selection the resistant colonies were expanded and incubated under reduced puromycin concentrations (3 µg/mL).

### Isolation and enrichment of HA-AP-EGF-R-expressing Sp2/0 cells

Magnetic activated cell sorting (MACS) was chosen to enrich puromycin-resistant cells that express and display the HA-AP-EGF-R on the cell surface. Therefore, puromycin-resistant cells were harvested. An aliquot of 5 × 10^6^ living cells was applied to the magnetic labeling procedure and incubated with the primary antibody (1 µg of a mouse anti-HA.11 monoclonal antibody per 1 × 10^6^ cells, Covance, MMS-101R, Munich) for 30 min at 4 °C. Further treatment was performed according to the manufacturer´s instruction by using the MACS Miltenyi Biotec Anti-Mouse IgG MicroBeads kit (130-048-401, lot number 5161103197, Miltenyi Biotec, Germany). Briefly, the cells were washed twice after labeling with the primary antibody by using PBS, pH 7.4, 0.5% BSA and 2 mM EDTA (MACS buffer) at 300 × g for 10 min. Following that, the magnetic labeling procedure was conducted by resuspending the cell pellet in 100 µL MACS buffer and adding 10 µL of the anti-mouse IgG microbeads suspension. After a 15 min incubation step at 4 °C the cells were washed twice by using MACS buffer. For the magnetic separation step the cell pellet was resuspended in MACS buffer and the suspension was added on a MACS column. Beforehand the MACS column was placed in the magnetic field of a suitable MACS separator. After the execution of the positive separation the cell suspension was washed once with MACS buffer and, following that, the cell pellet was resuspended in fresh growth culture media. The cells were cultured at 37 °C under 6% CO_2_.

### Antibody treatment of HA-AP-EGF-R-expressing Sp2/0 cells

The cells were treated with antibodies in suspension and washed by centrifugation. Antibodies and secondary reagents were diluted in PBS, pH 7.4, 0.5% BSA and 2 mM EDTA. As washing solution PBS, pH 7.4, 0.5% BSA and 2 mM EDTA was used.

### *In vitro* biotinylation of the surface receptor HA-AP-EGF-R by the biotin ligase BirA

A constant amount of stably transfected living cells was used for the *in vitro* biotinylation as described by Chen *et al*.^12^. The cell supernatant was discarded and the cells were washed twice with PBS supplemented with 5 mM MgCl_2_. Following that, the cells were incubated with a mixture consisting of 5 mM MgCl_2,_ 1 mM ATP, 10 µM Biotin and 0.789 µg per 1 × 10^6^ cells BirA (GeneCopoeia™ Source BioScience LifeSciences, Nottingham UK) for 30 min. To get rid of the reaction mixture, the cells were washed four times with PBS containing 5 mM MgCl_2_. To investigate the transfected cells by flow cytometry (FACSCalibur, BD), transfected and biotinylated cells were treated with PE-conjugated streptavidin (2 µg/mL; eBioscience, UK).

### Flow cytometry analysis of using the artificial receptor as bridge between the phenotype and genotype

To investigate whether the HA-AP-EGF-R is a suitable tool to retain a soluble antibody at the surface of the biotinylated transfected cells, a stable bridge was constructed between the receptor, an antigen and the antibody. Fluorescein was used as antigen which was bound to the surface as FITC-labeled SAV (2 µg/mL) (eBioscience, Hatfield UK) and the monoclonal mouse anti-fluorescein antibody B13-DE1^39^ was used as antibody to be bound. A PE-conjugated F(ab)_2_ fragment of a donkey anti-mouse IgG (12-4012, eBioscience, UK) diluted in PBS containing 0.5% BSA and 2 mM EDTA was used as indicator antibody.

### Fusion and HAT selection of HA-AP-EGF-R-containing Sp2/0 cells with spleen lymphocytes from ovalbumin immunized Balb/c mice

The hybridoma technique was used to analyze the cell surface expression behavior of the HA-AP-EGF-R fusion protein after fusion of a transfected Sp2/0 cell with spleen lymphocytes from an ovalbumin immunized Balb/c mouse. Immunization was performed according to the relevant national and international guidelines. The study was approved by the Ministry of Environment, Health and Consumer Production of the Federal State of Brandenburg (reference number V3-2347-A16-4-2012). Briefly, Balb/c mice (8–12 wks old) were immunized intraperitoneally with 100 µg ovalbumin (Sigma-Aldrich, USA) in 200 µL PBS supplemented with 50 µL Complete Freund´s Adjuvant (CFA). The fusion was conducted according to Köhler and Milstein^11^ with an electrofusion modification^40^. Briefly, spleen lymphocytes were mixed in a ratio of 3 to 1 with transfected myeloma cells that bear the artificial cell surface fusion protein and were washed three times with fusion buffer (125 mM NaCl, 5 mM KCl, 4 mM CaCl_2_, 2.5 mM MgCl_2_, 5 mM Tris-HCl pH 7.4, 0.2 µm). The cell pellet was dissolved in 200 µL fusion buffer and filled up to a final volume of 400 µL with 25% (w/v) PEG8000 in fusion buffer. The cell suspension was transferred into a fusion cuvette and placed into the electro fusion device. The cells were pulsed with 600 V for 25 ms. Three minutes post pulse the cell suspension was transferred into 37 °C pre-warmed full growth media supplemented with 20% (v/v) FCS and cultured for 5 h at 37 °C and 95% humidity under 6% CO_2_. Following that, HAT medium (hypoxanthine; 27.24 µg/mL, azaserine; 2 µg/mL and thymidine; 7.76 µg/mL) was added to the cell suspension. Finally the cell suspension was transferred into T75 flasks and further cultured at 37 °C and 95% humidity under 6% CO_2_. HAT selection was performed for 10 days. Growing hybrids were then checked for HA-AP-EGF-R surface receptor expression and secretion of ovalbumin-specific antibodies as described below.

### Isotype- and antigen-specific sorting of HA-AP-EGF-R+ hybridoma cells

Ten days after fusion HA-AP-EGF-R+ hybridoma cells were sorted either isotype- or antigen-specific. Therefore, the cells were first biotinylated *in vitro* with 0.5 mM biotin for 48 h at 37 °C and 6% CO_2_. After this, the cells were harvested and adjusted to a concentration of 1 × 10^6^ cells/mL. For the isotype-specific labeling the cells were washed twice with MACS buffer and incubated with a goat anti-mouse IgG specific antibody (115-005-164, lot number 128439, Dianova, Germany) conjugated to avidin (20 µg/mL) for 20 min at 4 °C. For the antigen-specific screening the cells were labeled with ovalbumin conjugated to avidin (Invitrogen, 20 µg/mL) for 20 min at 4 °C. After this incubation, the cells were washed again with 10 mL MACS buffer, the pellet was resuspended in 1 mL full growth cell culture media supplemented with 20% (v/v) FCS, 2 mM glutamine, 50 µM ß-mercaptoethanol and the cells were incubated for 3 h at 37 °C and 6% CO_2_ to allow antibody secretion and binding to the HA-AP-EGF-R construct. After incubation the cells were washed with 10 mL MACS buffer. The cell pellet was resuspended in 300 µL MACS buffer and 10 µg ovalbumin conjugated to Alexa Fluor 647 (Invitrogen, 2 mg/mL diluted in PBS) were added per sample (for 20 min at 4 °C). For panel I in Fig. 8a,b the cells were incubated only with 10 µg ovalbumin conjugated to Alexa Fluor 647. After this, the cells were washed twice with 10 mL MACS buffer, the pellet was resuspended in 500 µL MACS buffer supplemented with 3 µL 7-AAD (BD Biosciences, 100 µg/mL diluted in MACS buffer) and incubated for 15 min at 4 °C. The sample volume was adapted to 1 mL and analyzed by a flow cytometer (BD FACS Aria III, Becton Dickinson, Germany).

### Enzyme-linked immunosorbent assay (ELISA)

In order to detect the antigen-specificity and cross-reactivity of secreted antibodies an indirect ELISA was performed. Briefly, microtiter plates (greiner-bio one, Frickenhausen, Germany) were coated with 10 µg/mL ovalbumin and bovine serum albumin (BSA, Sigma-Aldrich, USA) in PBS (50 µL/well) overnight at 4 °C in a humid chamber. The wells were washed three times with tap water and blocked with PBS/NCS (5% neonatal calf serum, 50 µL/well) for 30 min at RT. After this, the wells were washed again and 50 µL/well culture supernatant from selected hybridomas were added and incubated for 1 h at RT. After washing a secondary goat anti-mouse IgG antibody conjugated to horseradish peroxidase (HRP; Lot number 129410, Dianova) was used for detection. The plates were incubated for 45 min at RT. Tetramethylbenzidine (TMB) solution (0.12 mg/mL TMB with 0.04% hydrogen peroxide in 25 mM NaH_2_PO_4_) was used as substrate. The reaction was stopped after 5 min with 1 M H_2_SO_4_. Optical density (OD) was measured at 450 nm with a reference of 630 nm.

### Statistics

Flow cytometry stainings were statistically validated by using BD CellQuest Pro software version 6.0 and BD FACSDiva software version 8.0. Graph Pad prism 6 was used for representing the ELISA data shown in Fig. 10.

## Data Availability

The data sets generated during and/or analysed during the current study are available from the corresponding author on reasonable request.
